# Reply letter to Battke et al.

**DOI:** 10.1038/s41431-021-00819-8

**Published:** 2021-02-17

**Authors:** Aida M. Bertoli-Avella, Christian Beetz, Najim Ameziane, Peter Bauer

**Affiliations:** CENTOGENE GmbH, Rostock, Germany

**Keywords:** Genomics, Genetics research

## To the Editor:

We appreciate the opportunity to reply to Battke et al., who commented on our paper, “Successful application of genome sequencing in a diagnostic setting: 1007 index cases from a clinically heterogeneous cohort” [1]. Genome sequencing (GS) was initially implemented in the clinical practice several years ago, but published experience from applications to large cohorts remains scarce. Our paper aimed at helping to close this gap.

In their letter, Battke et al. stated that our study is inappropriate for inferring statements about the diagnostic utility of GS. They also advocate for exome sequencing (ES) as the first-line test for rare disease genetic diagnostics. We largely disagree, and thus provide our line of thoughts below.

Battke et al. stated that they would have liked to have seen a ‘carefully designed’ study that compares ES and GS ‘fairly’ by analyzing a large cohort with both technologies in parallel. We fully agree with the value of such an approach and urge academic research groups to consider such pertinent projects. In a commercial routine diagnostic setting such as ours, however, implementation for a sufficient number of cases is not feasible for reasons of cost and time. Accordingly, our primary goal was not to directly compare both methods, but rather to describe our experience with GS—as clearly reflected in the title of our study.

Battke et al. went on to use our data for calculations of GS diagnostic yield and claimed that a non-GS approach would have been almost as successful in this respect. Most of these calculations are used incorrectly (use of ‘numbers of variants’ rather than ‘numbers of patients’). In saying that, we recognize that this could have possibly been due to not clearly presenting the data. More importantly, the calculations of Battke et al. focused on pathogenic (P) and likely pathogenic (LP) variants, while ignoring variants of uncertain significance (VUSs). This is crucial to note, as a large fraction of variants that are detected by GS, but are not (=have not been!) detected by other technologies, are expected to be VUSs, simply based on insufficient knowledge about frequencies in patients versus in controls (as of yet), lack of insight on the functional role of noncoding variants and, consequently, lack of classification guidelines for these type of genetic variation. We expect that future data sets, to be derived from further utilization of GS, will enable re-classification of many current VUSs. Considering this, a P/LP-restricted argumentation around diagnostic yield represents, at least partially, circular reasoning.

Lastly, Battke et al. elaborated on the ‘surprising result’ of a rarity of structural variants (SVs) in our cohort. While it remains unclear to us what their related criticism is, we would like to take this as an opportunity to re-emphasize a point that we believe constitutes one of the strongest arguments in favor of GS: the data, once generated, will be available for subsequent re-analysis by improved toolsets. As clearly stated, our study reports on real-life, GS-based diagnostics. It thereby restricts itself to findings that were made at the time of GS data generation. However, subsequent regular re-analysis of negative GS cases is continuously unraveling disease-relevant variants that were initially missed; additional diagnostic reports are consequently being issued. A recently published finding of ours, which did not even require a priori knowledge of a gene-disease association, exemplifies the success of such an iterative approach to GS-based diagnostics: A single exon deletion, i.e., a type of alteration likely to escape ES, was identified after improving the GS analytic algorithm such as to search for SV breakpoints [2]. For deep intronic and regulatory variants, the future improvement of tools will likely be of even greater relevance. An initially low fraction of ‘non-classical’ variants in GS can thus easily be increased, given appropriate diagnostic follow-up. Still, Battke et al. advocate that ES rather than GS should be the ‘method of first choice’. This promotion of an approach of ‘remaining blind’ despite having the tools ‘to be able to see’ does not align with offering the best existing solutions to rare disease patients.

We are all aware of, and excited about, the high dynamics in the development of genetic diagnostics. It is our strong belief that the choice of optimal tools relies on knowledge about the performance of these tools in real-life diagnostic settings. Our study added pertinent data to the literature, and we are convinced that the scientific community would welcome the publication of additional corresponding data sets.
